# Supplementary material for: A Comparative Study of Brain Injury Biomarker S100β During General and Spinal Anesthesia for Caesarean Delivery: A Prospective Study
Source: Medicina (Kaunas). 2025 Jul 30;61(8):1382. doi: 10.3390/medicina61081382 (PMC12387830; doi:10.3390/medicina61081382)
Supplement: Supplementary file 1 [file medicina-61-01382-s001.zip › medicina-3675712-supplementary.pdf]

**Supplement Table S1. Brain injury markers on Maternal post-surgery by symptoms**

| Variables | S100B | | P value |
|---|---|---|---|
| | **Mean** | **Standard Deviation** | |
| Symptoms that appeared within 2 hours after surgery | | | 0.024 |
| Nausea | 211.13 | 25.88 | |
| Vomiting | 214.16 | 13.13 | |
| Abdominal pain | 179.65 | 14.07 | |
| Headache | **215.14** | **27.75** | |
| Tremors | 206.11 | 47.98 | |
| Generalized weakness | 233.33 | 90.78 | |
| Tinnitus (ringing in the ears) | **279.36** | **147.38** | |
| Shortness of breath | 208.32 | 33.56 | |
| Blood loss | 203.32 | 36.16 | |
